# A comparison of nutritional value of native and alien food plants for a critically endangered island flying-fox

**DOI:** 10.1371/journal.pone.0250857

**Published:** 2021-05-19

**Authors:** Laura A. Pulscher, Ellen S. Dierenfeld, Justin A. Welbergen, Karrie A. Rose, David N. Phalen

**Affiliations:** 1 Sydney School of Veterinary Science, Faculty of Science, University of Sydney, Sydney, New South Wales, Australia; 2 Ellen S. Dierenfeld, LLC, St. Louis, Missouri, United States of America; 3 Nottingham Trent University, Southwell, United Kingdom; 4 Hawksbury Institute for the Environment, Western Sydney University, Richmond, New South Wales, Australia; 5 Australian Registry of Wildlife Health, Taronga Conservation Society Australia, Mosman, New South Wales, Australia; Kyoto University, JAPAN

## Abstract

Habitat loss and alteration are two of the biggest threats facing insular flying-foxes. Altered habitats are often re-vegetated with introduced or domestic plant species on which flying-foxes may forage. However, these alien food plants may not meet the nutritional requirements of flying-foxes. The critically endangered Christmas Island flying-fox (CIFF; *Pteropus natalis*) is subject to habitat alteration and the introduction of alien food plants, and therefore is a good model species to evaluate the potential impact of alien plant species on insular flying-foxes. In this study, we evaluated nutritional content of native food plants to determine how flying-foxes historically met their nutritional requirements. Furthermore, we compared the nutritional content of native and alien fruits to predict possible impacts of alien plants on insular flying-foxes. Native and alien fruits and flowers, and native foliage (leaves, petals, and petioles) commonly consumed by the CIFF were collected and evaluated for soluble sugars, crude protein, non-fiber carbohydrates, and nine minerals. Evaluation of native food plants suggests that flying-foxes meet energy requirements by consuming fruit and nectar. However, fruit and nectar are low in protein and essential minerals required for demanding life periods; therefore, flying-foxes likely supplement their diets with pollen and foliage. Thus, flying-foxes require a diverse array of plants to meet their nutritional requirements. Compared to native fruits, alien fruits contained significantly higher non-fiber carbohydrates, and this may provide an important energy source, particularly from species that bear fruit year-round. Median mineral concentrations in alien fruit species, however, were deficient compared to native fruits, suggesting major (or even seasonal) shifts in the proportion of alien species in the CIFF diet could lead to nutritional imbalances. This study confirms the need to quantify nutritional parameters in addition to feeding ecology when evaluating habitat quality to inform conservation actions that can be applied both locally and globally.

## Introduction

Flying-foxes (*Pteropus* spp.) are keystone species, maintaining habitat structure and diversity by providing significant ecosystem services such as pollination and seed dispersal [1]. These services are particularly important for sustaining the island ecosystems occupied by 80% of all flying-fox species; however, over half of all insular flying-foxes are classified as vulnerable, endangered, or critically endangered [2, 3]. On many islands, flying-foxes serve as the sole pollinator and seed disperser [4], and the loss of these species could prove catastrophic for the ecosystems that depend on these ecological services [5, 6]. Habitat loss and alteration are two of the biggest threats facing insular flying-foxes [2, 3]. Altered landscapes are often re-vegetated with introduced or domestic plant species, hereafter referred to as alien plants, that may be preferred by flying-foxes because of their increased availability, larger crop sizes, longer fruiting periods and increased palatability [7]. On island ecosystems where extensive native habitat loss or alteration has occurred, flying-foxes may shift their diet to predominantly incorporate alien food plants [8]. A study of food plants consumed by the Samoan flying-fox (*Pteropus samoensis*) illustrated how alien food plants were deficient in several nutrients including protein, copper, calcium, iron, and sodium [8]. Thus, it is not clear whether flying-foxes can meet their macro- and micronutrient requirements if they primarily consume alien plants.

Investigating the nutritional ecology of wild frugivorous bats is challenging because their nutritional requirements are unknown, it is difficult to determine all the plants that they feed on and the quality of each one that they consume, and there is limited data available on the nutrient composition of natural food sources. Additionally, nutritional recommendations for flying-foxes are extrapolated from data obtained from managed captive flying-foxes reliant on dietary ingredients largely restricted to agricultural fruit and formulated foods [9, 10]. This represents a considerable knowledge gap regarding how flying-foxes meet their nutritional requirements in the wild, particularly under increased demands required to sustain long-distance flying or during pregnancy and lactation.

Behavioral studies of frugivorous bats provide evidence that these animals require a variety of food plants to meet their nutritional requirements, including fruit, nectar, pollen, leaves and petioles [8, 9, 11–18]. Nutritional studies of frugivorous bat species have primarily focused on macronutrient content [12, 13, 19–21] and only a handful of studies reported micronutrient content of food plants consumed by flying-foxes [8, 14–16] and the short-nosed fruit bat (*Cynopterus sphinx*) [17], limiting our ability to accurately predict nutrient requirements. Nutrient analysis of native food plants would provide evidence for how flying-foxes met their nutritional requirements historically and how the introduction of alien food plants might impact the nutrition of insular flying-foxes.

An example of an island flying-fox that may be impacted by habitat alteration and the introduction of alien food plants is the critically endangered Christmas Island flying-fox (CIFF; *Pteropus natalis*). This species is exclusively found on Christmas Island, a small island in the Indian Ocean. Approximately 75% of Christmas Island is comprised of natural habitat; however, portions of the island have been extensively modified due to human activities. The northeastern part of the island has been developed for housing and associated infrastructure, and phosphate mining has occurred over 25% of the island, resulting in extensive land clearing on the eastern side of the island. Both development and mining have resulted in the introduction of alien plant species, for human consumption and post-mining restoration efforts, that are now food sources for the CIFF. Approximately 10% of the island contains alien plant species including agricultural plants such as mango (*Mangifera* spp.), banana (*Musa* spp.), soursop (*Annona muricata*), and breadfruit (*Artocarpus altilis*), as well as the Japanese cherry (*Muntingia calabura*) which is heavily planted in the first phase of reclamation efforts on mined land. The CIFF population declined from approximately 6,000 individuals in the 1980s to 2,000 individuals in 2007 [22–24]. The most recent population estimates are 3,800 individuals and now appears to have stabilized [18]. While the cause of this species’ decline is not known and may be multifactorial, possible drivers have been proposed including intoxication with the metal cadmium [25], predation by feral cats, stochastic weather events, possible nutritional stress, and reduced fecundity and health associated with feeding on alien plants [23]. Given that the CIFF is confined to a relatively small island, and the species of native and alien plants upon which they forage are relatively well-known [18, 26, 27], important opportunities exist to assess how the species utilizes its natural habitat to meet nutritional requirements and to analyze the potential impacts of alien plants on the overall health of insular flying-foxes. To this end, we examined the nutritional composition of native and alien food plants consumed by the CIFF to evaluate: 1) the historical utilization of native plants to meet flying-fox nutritional requirements, and 2) differences in nutrient content between alien and native food plants. Ultimately, we aim to predict possible positive or negative impacts of ecological and nutritional changes on the fecundity and health of insular flying-foxes.

## Methods

### Study location

Christmas Island is an Australian external territory located in the Indian Ocean, at 10°25’S and 105°43’E, approximately 380 km south of Java, Indonesia, and 1,500 km off the coast of Western Australia. The island has a land area of 135 km^2^ and is composed of tertiary limestone overlaying basalt volcanic rock that rises 361 m above sea level. Additionally, there are large phosphate deposits that are derived from seabird colonies that inhabit the island, creating a nutrition hotspot [28]. The climate is tropical with a temperature range of 22°C to 28°C and a high relative humidity (80–90%). There are distinct dry (July to October) and wet (November to June) seasons with an annual rainfall of 2 m [29]. Approximately 75% of the island is primary old-growth forest dominated by evergreen forest and semi-deciduous forest and scrub. The other 25% of the island has been cleared primarily for phosphate mining. Of this cleared area, approximately 13% is currently under lease for phosphate mining, 2% is reclaimed mining land currently undergoing rehabilitation by national parks, and the remaining 10% is regrown to varying degrees (S1 Table).

Apart from one epiphytic herb, no endemic plants are known to be extinct on Christmas Island; one endemic plant species, *Arenga listeri*, is listed as globally endangered but is widespread across the island [30]. Further, three fern species are listed as endangered or critically endangered nationally and are rare across the island but not likely consumed by the CIFF [30]. The old growth primary rainforest is essentially undisturbed and is minimally invaded by alien plant species. Most alien plant species are cultivated and are restricted to a small portion of the island (S1 Table). However, there are a few successful alien species that have naturalized and are common across the island including Japanese cherry, papaya (*Carica papaya*), guava (*Psidium guajava*), and alien *Syzygium* spp. [18]. Previous studies have identified 51 plant species in the CIFF diet including 21 spp. of alien fruits, 12 spp. of native fruits, 4 spp. of native leaves, 2 spp. of native petioles, 18 spp. of native flowers and 15 spp. of alien flowers [18, 26, 27].

### Collection and analysis of food plants

The plants analyzed in this study (S2–S8 Tables) represent the most common plant species on which the CIFF feeds [18, 26, 27]. For each plant, we aimed to collect 100 g (fresh weight) of material, including fruit, leaves, petals, and petioles consumed by the bats (hereafter referred to as ‘food plants‘). Samples were collected in May to August, 2018 and January to March, 2019 on Christmas Island, directly from trees or under trees at the stage during which the CIFF foraged. These months were targeted to reflect the seasonal phenology of food plants on Christmas Island with peak flower resources available in the dry season and peak fruit resources available in the wet season [18]. Specimens were collected and identified under the guidance of staff from Christmas Island National Park who have worked extensively on revegetation efforts in mining reclamation sites. Where possible 100 g of individual food plant samples (fruit, leaves, petals, or petioles) were collected from a single plant; if it was not possible to collect 100 g, samples (i.e., for Japanese cherries or *Ficus microcarpa*) were sourced from multiple trees of the same species within 100 m. In 3 instances (*Calophyllum inophyllum* n = 1; *Inocarpus fagifer* n = 2) 100 g could not be obtained from trees within 100 m; in these cases, samples were combined with materials from the same species found in closest proximity (all < 4 km). To account for potential differences in plant nutrient variability across the island, we collected replicates based on availability (mean n = 4; range = 1 to 14) of food plant samples from multiple locations across the island (see S2–S8 Tables for detailed information on replicate numbers for each food plant sample). Nectar (approximately 75 μL) was also opportunistically collected from select flowering plants into microcapillary tubes to determine sugar content, estimated immediately in the field using a handheld refractometer (Bacto Laboratories Pty Ltd, Australia) [31]. All plant samples were collected under the Australian Government Environment Protection and Biodiversity Conservation Regulations 2000 license to access biological resources in a commonwealth area for non-commercial purposes (Permit No AU-COM2018-414 & PA2018-00005).

Food plant samples were sliced, weighed, and frozen at -20°C until further processed. Within 90 days, samples were thawed, and where possible fruit juice was collected (~500 μL) into a graduated transfer pipette (Copan Italia SpA, Brescia, Italy) and soluble sugar content estimated with a handheld refractometer. Food plants were then desiccated in an oven at 55°C and weighed every 24 hours until two consistent dry weight measurements were achieved. Percent moisture was calculated by subtracting the final dry weight from the wet weight of each food plant. Food plant samples were then milled and placed into plastic bags with silica beads until transported for laboratory analysis; all laboratory analysis was conducted within 90 days of desiccating plants.

Twenty-gram subsamples of dried food plants were submitted to Dairy One Forage Lab (Ithaca, NY, USA) for nutrient analysis. Crude protein (CP), non-fiber carbohydrates (NFC), calcium (Ca), phosphorus (K), magnesium (Mg), potassium (K), sodium (Na), iron (Fe), zinc (Zn), copper (Cu), and manganese (Mn) were analyzed in all samples, and reported on a dry matter basis (DMB), whereas moisture (water) and soluble sugar concentrations were expressed as percentages on a wet (as-fed; AF) basis. The limits of detection for various assays were 0.1% for CP and NFC, 0.01% for Ca, K, Mg, K, and Na, 1 ppm for all trace metals (Cu, Fe, Mn, and Cu), and 1% soluble sugar for the refractometer. In addition, energy density was calculated for all food plants. Approximate values were obtained from previously published studies [8, 12, 16, 32], the Australian Food Composition Database [33], or the U.S. Department of Agriculture FoodData Central Database [34] for crude fat (CF; S2 Table). For any plant species where approximate CF values could not be found, a proxy value of 2.4% DM was used based on recommendations by Dairy One for CF in miscellaneous forages (S2, S4 and S6 Tables). Energy density was calculated using CF, CP, and NFC (all AF) and converted to a DMB (kJ/g DMB).

### Statistical analysis

All statistical analyses were performed in R version 3.6.0 for Windows [35] using the package Factoextra (version 1.0.7) [36]. Significance was set at α < 0.05. Samples of insufficient size, or nutrients with results below analytical detection limits were classified as NA, and not included in the subsequent statistical evaluation. To examine the pattern of variation between species of 1) native fruits and native foliage (native leaves, petals, and petioles) and 2) native fruits and alien fruits; macronutrient (NFC, CP) and micronutrient (Ca, Mg, Na, P, Ca:P, K, Cu, Fe, Mn, and Zn) data were entered into a principle components analysis (PCA). Prior to analysis, normality was determined using a Shapiro-Wilk test and assessment of Q-Q plots and histograms [37]. Data were determined to be non-normally distributed and were transformed using a box-cox transformation, excepting NFC and Cu that were transformed using ordered quantile normalization prior to PCA analysis.

Median nutritional attributes and energy density of 1) native fruits and leaves and 2) native and alien fruits were further compared with a Wilcoxon Rank Sum Test. As data were non-normally distributed, replicate medians for each nutrient were calculated for each species and used for all pairwise comparisons; these data are provided as S2–S8 Tables. Data were combined into defined groups (alien fruits, native fruits, or native leaves), median values were calculated for each category, and a Wilcoxon Rank Sum Test was run. To manage type 1 errors a Bonferroni correction for multiple testing was applied.

## Results

One-hundred and twenty five food plant samples were collected, including alien fruits (n = 55, from 17 spp., and 12 families), native fruits (n = 21, from 8 spp., and 7 families), native leaves (n = 37, from 3 spp., and 3 families), native petioles (n = 3 from 1 sp.), native flower petals (n = 1 from 1 sp.), and nectars from alien (n = 4, from 4 spp., and 3 families) and native (n = 4, from 2 spp., and 2 families) flowers. This included 81% of alien fruit spp., 50% of native fruit spp., 75% of native leaves, 50% of native petioles, and 20% of alien flowers and 17% of native flowers that have previously been observed in the CIFF diet [18, 26, 27]. Additionally, 2 species of native pandan fruits (*Pandanus christmatensis* and *P*. *elatus*) were collected as they are considered likely to appear in the CIFF diet but have not been directly observed [18].

Fruit juice was collected from 14 spp. of alien fruits (n = 45 from 8 families) and 2 spp. of native fruits (n = 6 from 2 families). Of these species, adequate levels of juice could not be collected from six fruit samples: soursop (n = 1), banana (n = 1), Japanese cherry (n = 2), and native *C*. *inophyllum* fruits (n = 2), and were not included in subsequent analysis for sugar content calculations. Furthermore, as juice could not be extracted from native foliage, pairwise comparisons of sugar content were not computed for native fruits and leaves. Of food plants collected, 117 samples were submitted for nutritional analysis. Three samples (n = 1 papaya, n = 1, sapodilla (*Manilkara zapota*), and n = 1 *Manilkara* spp.) had copper levels that were below the detection limit and were not included in subsequent analyses.

**Fig 1:**
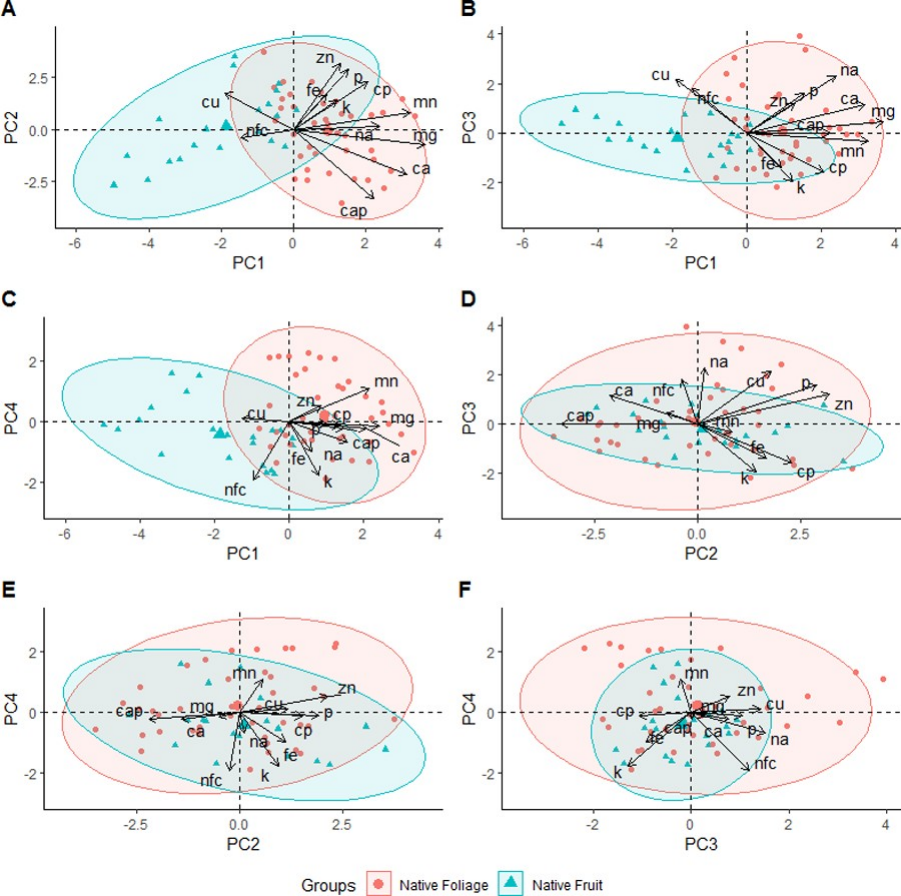
Results of the PCA analysis with 95% ellipses comparing nutrients for native fruits (circle) and foliage (triangle) consumed by the Christmas Island flying-fox (*Pteropus natalis*). Results of the PCA for A) principal component (PC) 1 and 2, B) PC 1 and 3, C) PC 1 and 4, D) PC 2 and 3, E) PC 2 and 4, and F) PC 3 and 4.

The PCA analysis for native fruits and foliage (leaves, petals, and petioles) identified four principal components (PC) with eigenvalues ≥ 1 that explained 73.7% of the variation (Table 1). Principal component 1 was loaded heavily and positively for Ca, Mg, and Mn. Principal component 2 was loaded heavily and positively for P and Zn but heavily and negatively for Ca:P. Principal component 3 was loaded heavily and positively for Na and Cu and PC 4 was loaded heavily and negatively for NFC and K. A plot of the factor loadings for each species of native fruits and foliage sampled did not reveal full separation of native food plants along any axis, although native foliage tended to score higher on PC 1 and 4 compared to native fruits (Fig 1; detailed figures in S1 Fig). Pairwise comparisons revealed native leaves contained higher median concentrations of Ca (W = 0, p = 0.02), Mg (W = 2, p = 0.05), and Mn (W = 1.5, p = 0.04) but lower energy density (W = 22, p = 0.05) (Table 2). However, none of the variables remained significant after applying a Bonferroni correction (|p| > 0.05).

**Table 1:** Factor loadings from principal component analyses.

	Native Fruits and Foliage				Native and Alien Fruits		
	PC1	PC2	PC3	PC4	PC1	PC2	PC3
**Eigenvalue**	3.37	2.74	1.49	1.23	4.79	2.06	1.46
**Percent variance (%)**	28.1%	22.9%	12.5%	10.3%	39.9%	17.2%	12.2%
**Cumulative variance (%)**	28.1%	51.0%	63.4%	73.7%	39.9%	57.1%	69.3%
**Loadings**							
Non-Fiber Carbohydrates	-0.187	-0.056	0.347	-0.612	-0.279	0.235	-0.307
Crude Protein	0.265	0.327	-0.306	-0.041	0.337	0.273	-0.156
Calcium	0.405	-0.306	0.223	-0.074	0.313	-0.414	-0.138
Magnesium	0.468	-0.107	0.085	-0.045	0.317	0.066	-0.440
Sodium	0.305	0.025	0.445	-0.214	0.146	-0.367	-0.130
Phosphorus	0.196	0.413	0.304	-0.030	0.368	0.237	-0.049
Calcium:Phosphorus	0.286	-0.474	0.002	-0.070	0.124	-0.615	-0.111
Potassium	0.156	0.202	-0.375	-0.568	0.173	0.265	-0.480
Copper	-0.244	0.253	0.411	0.034	0.252	0.125	0.576
Iron	0.118	0.240	-0.268	-0.309	0.332	-0.092	0.039
Manganese	0.418	0.120	-0.065	0.345	0.336	0.148	0.112
Zinc	0.165	0.459	0.229	0.164	0.333	0.098	0.248

**Table 2:** Nutrient composition for native fruits [median (range)], alien fruits [median (range)], and native leaves [median (range)], petioles [mean (sd)], and petals consumed by the Christmas Island flying-fox (*Pteropus natalis*).

	Food Plant Category						Nutrient Requirements for Laboratory Rats^g^	
Nutrient^a^	Native Fruits (n = 21, 8 spp., 7 families)	Alien Fruits (n = 55, 17 spp., 12 families)	Native Leaves (n = 37, 3 spp., 3 families)	Native Petioles (n = 3, 1 sp. & family)	Native Petals (n = 1, 1 sp. & family)	Fruit bat dietary nutrient recommendation^f^	Growth	Reproduction
***Proximate Components***								
Soluble Sugar (%)	9.8 (5.5–14)^b^	10.0 (1.0–20)^c^	n.a.^e^	n.a.	n.a.	n.a.	n.a.	n.a.
Moisture (%)	69.7 (42–86)	78.7 (50–93)	72.6 (66–77)	70.4 (5.5)	79.9 (.)	n.a.	n.a.	n.a.
CP (%)	7.9 (3.3–18)	5.7 (2.4–10)	13.6 (12–14)	5.5 (0.7)	20.5 (.)	6.5–18.3	15.0	15.0
NFC (%)	39.6 (11–50)	66.0 (41–79)*	34.0 (15–39)	39.5 (7.2)	34.9 (.)	n.a.	n.a.	n.a.
Energy density (kJ/g)	11.5 (7.1–31)	13.4 (8.2–33)	9.2 (6.2–9.4)	8.4 (1.1)	10.2 (.)	-		
***Minerals***								
Ca (%)	0.45 (0.3–0.9)	0.23 (<0.1–0.8)	1.21 (1.2–2.1)	2.04 (0.2)	0.32 (.)	0.63–0.85	0.50	0.63
Mg (%)	0.11 (0.1–0.3)	0.08 (<0.1–0.3)	0.22 (0.2–0.4)	0.56 (0.2)	0.16 (.)	0.09–0.17	0.05	0.06
Na (%)	0.18 (<0.1–0.6)	0.04 (<0.1–0.5)	0.16 (0.1–0.9)	0.51 (0.3)	0.02 (.)	0.06–0.21	0.05	0.05
P (%)	0.21 (0.1–0.4)	0.17 (<0.1–0.4)	0.24 (0.2–0.3)	0.76 (0.2)	0.36 (.)	0.52–0.61	0.30	0.37
K (%)	1.16 (0.6–2.7)	1.17 (0.7–1.9)	1.24 (1.2–1.6)	1.19 (1.0)	2.11 (.)	0.90–1.16	0.36	0.36
Cu (mg/kg)	10 (7.0–13)	6.0 (1.5–114)^d^	8.0 (6.0–9.0)	7.3 (0.6)	9.0 (.)	7–15	5	8
Fe (mg/kg)	101 (25–451)	31 (15–289)	57 (52–72)	26 (6.0)	281 (.)	140–411	35	75
Mn (mg/kg)	14 (6.0–26)	8.0 (3.0–48)	26 (25–60)	41 (23)	18 (.)	30–84	10	10
Zn (mg/kg)	22 (13–37)	12 (3.0–857)	27 (17–31)	54 (15)	70 (.)	29–90	12	25

All data, except moisture and soluble sugar content, are presented on a dry matter basis. A Wilcoxon rank sum test followed by a Bonferroni correction was used to evaluate statistical differences between nutrients in native and alien fruits.

*p≤0.05.

^a^Abbreviations for nutrients are as follows: crude protein (CP), non-fiber carbohydrates (NFC), calcium (Ca), magnesium (Mg), sodium (Na), phosphorus (P), potassium (K), copper (Cu), iron (Fe), manganese (Mn), and zinc (Zn).

^b^Only 2 native fruit spp. had adequate quantities of juice for analysis.

^c^Only 14 alien fruit spp. had adequate quantities of juice for analysis.

^d^One alien fruit sp. was under the limit of detection and was not included in the analysis for Cu.

^e^n.a. = not analyzed or not available.

^f^Recommendations from the Fruit Bat Husbandry Manual from the American Zoo and Aquarium Association Chiropteran Taxon Advisory Group [9].

^g^Laboratory rat nutrient requirements from the National Research Council (US) Subcommittee on Laboratory Animal Nutrition [38].

**Fig 2:**
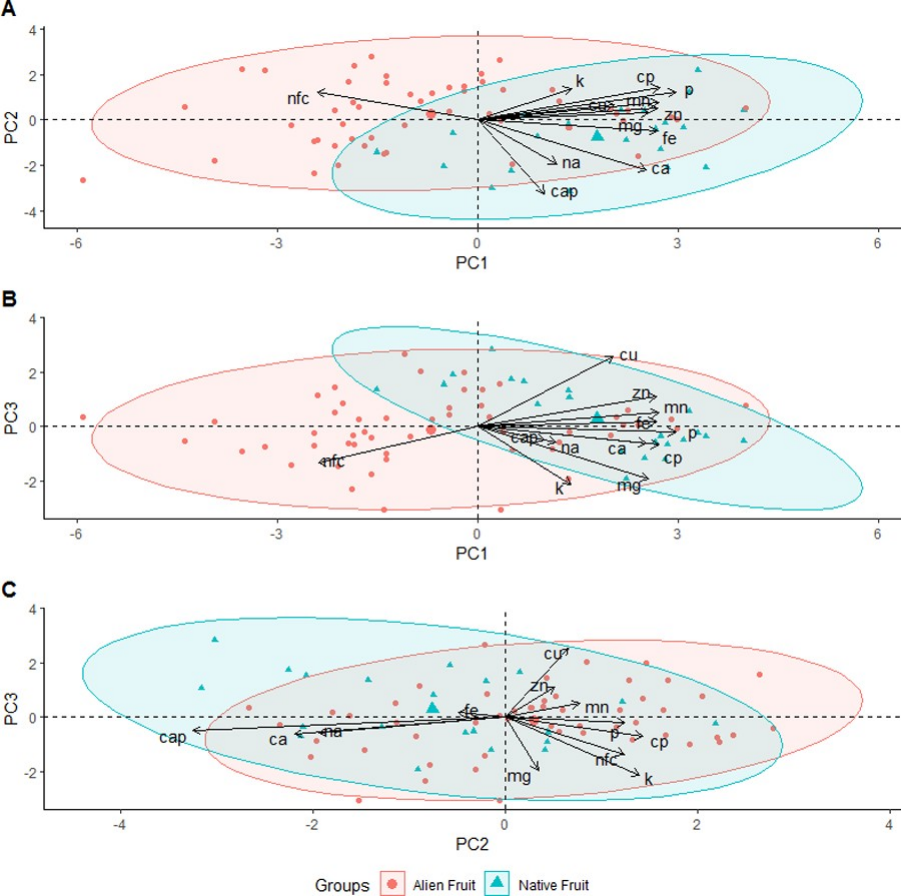
Results of the PCA analysis with 95% ellipses comparing nutrients for alien fruits (circle) and native fruits (triangle) consumed by the Christmas Island flying-fox (*Pteropus natalis*). Results of the PCA for A) principal component (PC) 1 and PC 2, B) PC 1 and 3, and C) PC 2 and 3.

The PCA analysis for native and alien fruits identified three PC’s with eigenvalues ≥ 1 that explained 69.3% of the variation (Table 1). Principal component 1 was loaded moderately and positively for CP, Ca, Mg, P, Fe, Mn, and Zn. Principal component 2 loaded heavily and negatively for Ca and Ca:P. Principal component 3 loaded heavily and positively for Cu but heavily and negatively for Mg and K. A plot of the factor loadings for each species of fruit sampled did not reveal full separation of native and alien fruits along any axis, although native fruits tended to score higher on PC 1 and 3 but lower on PC 2 compared to alien fruits (Fig 2; detailed figures in S2 Fig). Pairwise comparisons of alien and native fruits revealed median concentrations of alien fruits contained lower levels of Ca (W = 28, p = 0.02), Cu (W = 25.5, p = 0.02), Fe (W = 23.5, p = 0.01), and Na (W = 35, p = 0.05) but higher NFC (W = 130, p < 0.001) compared to native fruits (Table 2). However, only NFC (p = 0.004) differences remained significant after a Bonferroni correction. Although not significantly different, Fe was more than 3—fold higher in native species in comparison with alien species (Table 2); one alien species, mangosteen (*Garcinia xanthochymus*), and various native species (*Terminalia catappa*, *I*. *fagifer*, *F*. *microcarpa*, and *P*. *christmatensis*) also contained considerably higher Fe concentrations than other fruits (S3 and S5 Tables). Additionally, although not analyzed statistically due to insufficient sample size, average soluble sugar content of nectars from native flowers (14.0%) was similar to that of three of four alien flowers (14.8%), with passionfruit (*Passiflora* spp.) a notable exception (47.0% (n = 1)) with extremely high soluble sugar concentrations (S8 Table).

In general, food plant nutrients fell within the diet recommendations for laboratory rats and other *Pteropus* spp. as outlined by the American Zoo and Aquarium Association (AZA) chiropteran advisory group (Table 2). However, median concentrations of protein for alien and native fruits and petioles were lower than levels required for growth and reproduction of laboratory rats. Furthermore, Ca, Na, Cu, Fe, Mn and Zn were lower for alien fruits compared to native fruits, foliage, and AZA and laboratory rat recommendations. Additionally, native *Macaranga tanarius* petioles and *Erythrina variegata* petals were the only food plants analyzed with concentrations of P within the AZA and laboratory rat recommendations (S7 Table).

## Discussion

### How did flying-foxes meet their nutritional requirements with native food plants?

Evaluation of native food plants consumed by the CIFF in our study suggest that the bulk of energy is derived from native fruits (median 10.5 kJ/g DM), particularly those that are high in fat such as *Planchonella nitida* (12.6 kJ/g DM), *C*. *inophyllum* (11.6 kJ/g DM), and *I*. *fagifer* (11.4 kJ/g DM) and pandan fruit if foraged on (*P*. *christmatensis* 30.5 kJ/g DM and *P*. *elatus* 24.7 kJ/g DM), as well as nectar (median 0.03 kJ/mL) from native flower species. This is analogous to other studies that suggest flying-foxes fuel their energy demands with sugars from nectars or fruits [39]. Food availability on Christmas Island is highly seasonal with peak flower resources available in the dry season (August–December) and peak fruit resources available during the wet season (December–April) [18]. Thus, CIFF likely meet their energy requirements predominantly from flowers in the dry season and switch to fruits in the wet season. Some native fruit species, specifically *Ficus* spp. and *P*. *nitida*, are also available during the dry season, providing important energy resources for CIFF during the transition from the wet to dry season as has been previously suggested [18]. Native fruits and flower species are calorie rich but are generally low in protein and essential minerals which are necessary to support growth, gestation, and lactation in mammals. Therefore, flying-foxes likely supplement their diets with native leaves, petioles, petals and pollen from native flowers to meet protein and essential mineral requirements.

Previous studies suggest that frugivorous bat species can survive on low protein diets, and that a diet consisting of 4–6% protein is sufficient for the maintenance of captive *Pteropus* spp. [9]. These requirements are suspected to increase for growth and reproduction but have not been experimentally determined in flying-foxes. Results from our study indicate that median protein in native fruits (7.93% DMB) is sufficient for physiological maintenance of CIFFs but may not fully support growth and reproduction. However, CIFFs also forage on the leaves, petals, and petioles of native plants and pollen from native flower species [18] and this likely increases protein intake. Similarly, other studies of frugivorous bats have reported the consumption of insects, pollen, leaves, and petals suggesting that other species also depend on these food sources to meet their protein requirements [9, 11, 13, 14, 16, 18, 40, 41]. Compared to native fruits, protein concentrations in native leaves (median 13.6% DMB) and petals (20.5% DMB) are within suggested protein levels recommended for growth and reproduction of laboratory rats [38], suggesting CIFF likely utilize native leaves to meet elevated protein requirements.

Pollen from native flowers may also supplement protein needs of CIFFs. Due to quarantine restrictions, we were unable to determine nutritional values of pollen consumed by the CIFF. However, previous studies of pollens from similar plant genera and families have identified protein content ranging from 13.3–34.7% DMB [42]. As the percentage of protein that is in pollen is highly conserved among plant genera and families [43], it can be assumed that pollen might also be an important source of protein for the CIFF. Pollen digestion efficiency by the CIFF is unknown, however, preliminary investigation suggests 50–75% of native pollen grains are digested by CIFF (unpublished data). This proportion falls within the range of pollen digestion (27–86%) reported for other frugivorous bat species [43–45]. Further, amino acid requirements have not been determined for flying-fox species; however, studies of Australian honeybee collected pollens from similar plant genera and families suggest these pollens were sufficient for physiological maintenance but not growth and reproduction of laboratory rats [38, 42]. Thus, pollen can be an important supplementary source of essential amino acids and protein for the CIFF, however further detailed studies of pollen nutrient composition and digestion are necessary to fully understand the nutritional significance of pollen in the CIFF’s diet.

Mammals also require sufficient Ca and P to meet increased nutritional demands for growth and reproduction [38, 46]. While specific requirements have not been determined for chiropterans, evaluation of milk from flying-foxes suggest higher concentrations of Ca (0.84–0.94% DMB) and P (0.60–0.62% DMB) are necessary to meet the nutritional demands of growing flying-fox pups [47]. If milk composition is representative of need, *F*. *microcarpa* fruit, and all native leaves and petioles had sufficient Ca to support growth and reproduction of flying-foxes. Similar findings have been reported from studies of native figs and leaves consumed by flying-foxes in American Samoa [8, 14, 16] and the short-nosed fruit bat [17]. Native *M*. *tanarius* petioles were the only plant in this study that displayed adequate percentages of P in comparison to recommendations for captive *Pteropus* spp. [9]. However, *I*. *fagifer* fruit and *E*. *variegata* petals fall within the ranges of P recommended for sustaining growth and reproduction of laboratory rats [38]. It is expected that flying-foxes would be able to meet required P concentrations with these native plant species, however, further studies are required to confirm this.

A potentially important finding in this study was the high Fe concentrations in native fruits (median 171 mg/kg; range 38–451 mg/kg DMB). For captive flying-foxes, diets are suggested to contain less than 100 mg/kg Fe [48], due to the susceptibility of Egyptian fruit bats (*Rousettus aegyptiacus*) to iron storage disease (ISD) in captivity [49–51]. Excessive Fe storage has been previously documented in flying-foxes, but not associated with clinical disease [51]; therefore flying-foxes may be less susceptible to ISD or have other mechanisms to control Fe metabolism. In a separate study [25], hepatic Fe concentrations of two CIFFs were considerably lower than values reported in Egyptian fruit bats with ISD [49, 51]. High urinary Fe concentrations are reported in CIFFs (median urine Fe 564 μg/g creatinine) [25] and grey-headed flying-foxes (*P*. *poliocephalus*; median urine Fe 256 μg/g creatinine) [52], suggesting that renal excretion is an important route for maintaining Fe homeostasis in flying-foxes. It is also possible that Fe concentrations in native fruits have limited bioavailability due to naturally occurring tannins and phytates [53, 54] or CIFFs may build up Fe stores during certain times of the year as has been documented in wild European starlings (*Sturnus vulgaris*) [53, 55] to compensate for seasonal dietary limitations. These findings suggest that Fe metabolism in flying-foxes needs to be investigated in more detail.

Regarding other trace minerals, Mn concentrations for all native fruits were below the nutritional recommendations for captive Pteropodids [9], but mostly sufficient to support growth and reproduction of laboratory rats [38]. All native foliage contained adequate Mn as recommended for both *Pteropus* spp. and laboratory rats, suggesting CIFFs are able to meet Mn requirements through consumption of native foliage. Excepting *F*. *microcarpa* leaves, all foliage and fruits from *T*. *catappa*, *I*. *fagifer*, and *P*. *nitida* contained Zn concentrations within the recommendations for both captive Pteropodids and laboratory rats [9, 38]. In addition, most native fruits and all native leaves and petioles contained sufficient levels of Cu, Mg, Na, and K to meet nutritional recommendations for *Pteropus* spp. and laboratory rats [9, 38], suggesting these are not limiting nutrients in this population. Of all the native food plants assessed in this study, no single plant species provided all the required nutrients necessary for growth and reproduction of laboratory mammals [38]. However, fruits from *F*. *microcarpa*, *I*. *fagifer*, and *T*. *catappa* and foliage overall contained sufficient concentrations of most measured nutrients, suggesting these native plants are particularly important for CIFFs. Data from native food plants of the CIFF provide evidence that a diversity of food plants is required to sustain the CIFF population. Flying-foxes with similar dietary preferences likely have similar nutritional requirements, suggesting that the conservation of large tracts of native habitat and botanical diversity is paramount in the conservation of flying-foxes worldwide.

### How does the introduction of alien food plants alter the nutritional content of the diet of flying-foxes?

In areas with extensive habitat loss or alteration, the introduction of nutritionally deficient alien food plants could impair the ability for insular flying-foxes to meet their nutritional requirements [8]. This study found that alien fruits contained significantly more carbohydrates compared to native fruits. Therefore, flying-foxes may increasingly forage on alien fruits or flowers due to their high soluble sugar concentrations, making it easier to meet their energy requirements in a shorter amount of time. However, median energy density of alien fruits (13.4 kJ/g) and flowers (0.03 kJ/mL) did not significantly differ from native fruits (11.5 kJ/g) and native flowers (0.03 kJ/mL). Therefore, foraging on alien fruits is not likely due to energy content alone as has been previously suggested [8]. Instead, CIFFs may forage on alien fruits because they are often larger and some, including papaya and Japanese cherry, fruit year-round on Christmas Island [18], even when other food may be scarce.

Although protein concentrations did not significantly differ between native and alien fruits, no alien fruits from this study contained sufficient protein for growth and reproduction of laboratory rats [38]. However, most alien fruits did contain sufficient protein to support maintenance of captive *Pteropus* spp. [9]. Pollen from alien flower species is another potential source of protein; however, previous studies of the CIFF diet reported that less than 10% of pollen identified on CIFF was from alien flower species [18], and the digestibility of alien pollen is unknown. Therefore, alien flower pollen is unlikely an important protein resource for the CIFF but further studies assessing this are required.

Macromineral concentrations for alien fruits in this study were lower compared with native fruits, with the exception of K, corroborating findings by Nelson et al. [8]. Furthermore, no alien fruits collected provided adequate concentrations of all essential minerals. While median Ca concentration in alien fruits was one-half that of native fruits, some individual species (i.e., breadfruit, Japanese cherry and mangosteen) did have similar mineral profiles to native *F*. *microcarpa*, suggesting they may be important alien food sources for the CIFF particularly during reproduction and lactation. Furthermore, P concentrations were similar between alien and native fruit species, but Japanese cherry was the only alien fruit with adequate concentrations to support nutritional requirements of other *Pteropus* spp. [9] and laboratory rats during growth and lactation [38]. Median Na concentrations in alien fruits were less than one quarter of those compared to native fruits, but again, individual (n = 7) alien fruits contained sufficient Na to support growth and reproduction of laboratory rats [38]. Neither Mg nor K appeared limiting in alien fruits.

With respect to trace minerals, and corroborating findings from Nelson et al. [8], most alien fruits in this study had similar or much lower levels of Cu, Fe, Mn, and Zn concentrations compared to native fruits. Mangosteen was the only notable exception with relatively high Fe (289 mg/kg) and excessive Zn (857 mg/kg) concentrations. Unlike native plants, domesticated plants have been bred for human palatability and as such may not have naturally occurring tannins and phytates. Therefore, it is possible that flying-foxes could absorb excessive amounts of Fe if they preferentially forage on this plant. The extremely high Zn concentrations (857 mg/kg) found in mangosteen were also concerning. Previous studies with laboratory rats report that Zn concentrations > 250 mg/kg can induce Cu deficiency, particularly if Cu intake is minimal; furthermore, excess Zn consumption can result in haemolysis and impact nervous system and kidney function [38]. However, it is unlikely that Fe and Zn toxicity is a problem in the CIFF population, since mangosteen is not prevalent across the island and is likely diluted in the diet of the CIFF. Additionally, preliminary investigations of liver samples from two CIFFs found liver Fe and Zn concentrations [25] were within the normal range reported for mainland Australian flying-foxes in the wild [52], as well as other frugivorous bat species [56].

Our study adds to the existing literature demonstrating that alien food plants can be deficient in certain nutrients; therefore, a major shift in the consumption of alien food plants could lead to possible decreased fecundity and/or overall health of insular flying-foxes. However, some alien food plants, specifically guava, Japanese cherry, mangosteen, passionfruit, and *S*. *jambos*, had similar nutritional profiles to that of native plants and might be important alien food plants for flying-foxes. Japanese cherry, in particular, seems to be the most nutrient rich alien food plant for the CIFF as it had the second highest crude fat (2.3%) and energy density (15.2 kJ/g) of all alien fruits assessed. Furthermore, it was sufficient in all other essential minerals, apart from Na and Fe. Japanese cherry is highly preferred by the CIFF [18] and is commonly planted in rehabilitated areas widespread across the island. However, Japanese cherries are low growing trees that could increase the susceptibility of the CIFF to predation by feral cats. Eradication efforts of feral cats on Christmas Island are ongoing and we suggest these efforts continue, particularly within dense stands of Japanese cherries.

How the CIFF utilizes its foraging landscape is still being investigated. Studies of the CIFF report that native flowers are preferred to alien flowers, but alien fruits, specifically Japanese cherry, banana, and papaya, topped the list for the most frequently occurring fruit resources in the CIFF diet [18], however more in depth studies are required to fully understand the electivity of CIFF for native and alien plants. It is possible that flying-foxes primarily forage on alien fruits or nectar producing plants early in the evening to sustain their energy requirements in search of more nutritious native food plants throughout the night. Furthermore, it is not entirely clear if the use of alien food plants differs by sex or age group and how this differs across seasons. Considering the increased demands during pregnancy and lactation in females, and growth of juveniles, it would be valuable to determine the percentage of time CIFFs spend foraging on alien plants in comparison to native plants in future studies.

## Conclusion

This study adds to the sparse knowledgebase on the nutritional content of plants consumed by flying-foxes and informs the understanding of nutrition in other flying-fox populations that consume similar foodstuffs. In addition, our study provides evidence that a diversity of food plants and parts are required to sustain flying-fox populations, thus supporting the notion that preservation of complex foraging habitats is paramount in the conservation of flying-foxes worldwide [8, 11, 13, 14, 16]. Further, this study demonstrates that some alien fruits are deficient in important micronutrients that if primarily foraged on, could make it difficult for flying-foxes to meet their nutritional requirements. Nonetheless, some alien plants had similar nutritional profiles to that of native plants and may be important alien food plants for flying-foxes. In the case of the CIFF, and other insular species where habitat destruction has occurred, revegetation plans should focus on native food plants, or, if necessary, alien food plants with nutritional profiles similar to those of native species to provide adequate nutrient resources for flying-foxes throughout the year. Further studies are necessary to determine the quantities of and preferences for alien versus native foods consumed by the CIFF, as well as the impact of pollen in the diet. Foraging and nutritional ecology are critically important to natural resource management and species survival, particularly on island ecosystems where resources are often finite and altered and therefore should be a research priority for the sustained conservation of insular flying-fox species worldwide.

## Supporting information

S1 Fig Detailed results of the PCA analysis with 95% ellipses comparing nutrients for native fruits (circle) and foliage (triangle) consumed by the Christmas Island flying-fox (*Pteropus natalis*). Results of the PCA for A) principle component (PC) 1 and 2, B) PC 1 and 3, C) PC 1 and 4, D) PC 2 and 3, E) PC 2 and 4, and F) PC 3 and 4.
(JPEG)

S2 Fig Detailed results of the PCA analysis with 95% ellipses comparing nutrients for alien fruits (circle) and native fruits (triangle) consumed by the Christmas Island flying-fox (*Pteropus natalis*). Results of the PCA for A) principle component (PC) 1 and PC 2, B) PC 1 and 3, and C) PC 2 and 3.
(JPEG)

S1 Table Classification of habitat types on Christmas Island available from Geoscience Australia <https://d28rz98at9flks.cloudfront.net/82430/CI_vegetation_and_clearing_map.zip>. (XLSX)

S2 Table Macronutrient composition for alien fruits [median(range)] consumed by the Christmas Island flying-fox (*Pteropus natalis*). All data, except dry matter and energy density, are presented on a as fed basis.
(XLSX)

S3 Table Micronutrient composition for alien fruits [median(range)] consumed by the Christmas Island flying-fox (*Pteropus natalis*). All data are presented on a dry matter basis.
(XLSX)

S4 Table Macronutrient composition for native fruits [median(range)] consumed by the Christmas Island flying-fox (*Pteropus natalis*). All data, except dry matter and energy density, are presented on a as fed basis.
(XLSX)

S5 Table Micronutrient composition for native fruits [median(range)] consumed by the Christmas Island flying-fox (*Pteropus natalis*). All data are presented on a dry matter basis.
(XLSX)

S6 Table Macronutrient composition for native foliage [median(range)] consumed by the Christmas Island flying-fox (*Pteropus natalis*). All data, except dry matter and energy density, are presented on a as fed basis.
(XLSX)

S7 Table Micronutrient composition for native foliage [median(range)] consumed by the Christmas Island flying-fox (*Pteropus natalis*). All data are presented on a dry matter basis.
(XLSX)

S8 Table Median (range) soluble sugar content and energy density for native and alien flower nectar consumed by the Christmas Island flying-fox (*Pteropus natalis*). All data is on an as fed basis.
(XLSX)

S9 Table Raw data for all native and alien fruits, leaves, petioles, and petals consumed by the Christmas Island flying-fox (*Pteropus natalis*). (XLSX)

S10 Table Raw data for all native and alien flowers consumed by the Christmas Island flying-fox (*Pteropus natalis*). (XLSX)
