# Supplementary figures and images for: A comparison of nutritional value of native and alien food plants for a critically endangered island flying-fox
Source: PLoS One. 2021 May 19;16(5):e0250857. doi: 10.1371/journal.pone.0250857 (PMC8133443; doi:10.1371/journal.pone.0250857)

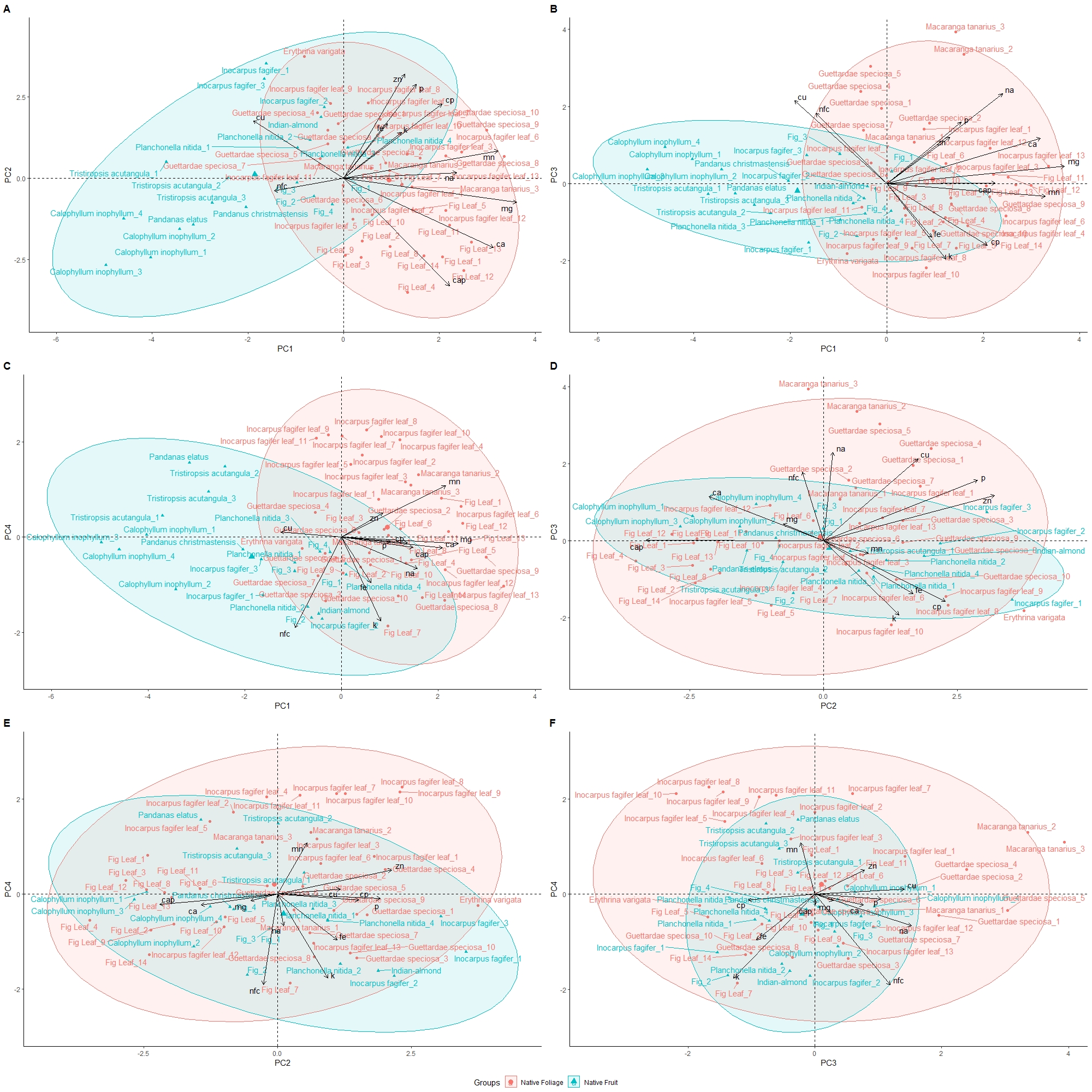

Supplement: S1 Fig — Results of the PCA for A) principle component (PC) 1 and 2, B) PC 1 and 3, C) PC 1 and 4, D) PC 2 and 3, E) PC 2 and 4, and F) PC 3 and 4. (JPEG) [file pone.0250857.s001.jpeg]

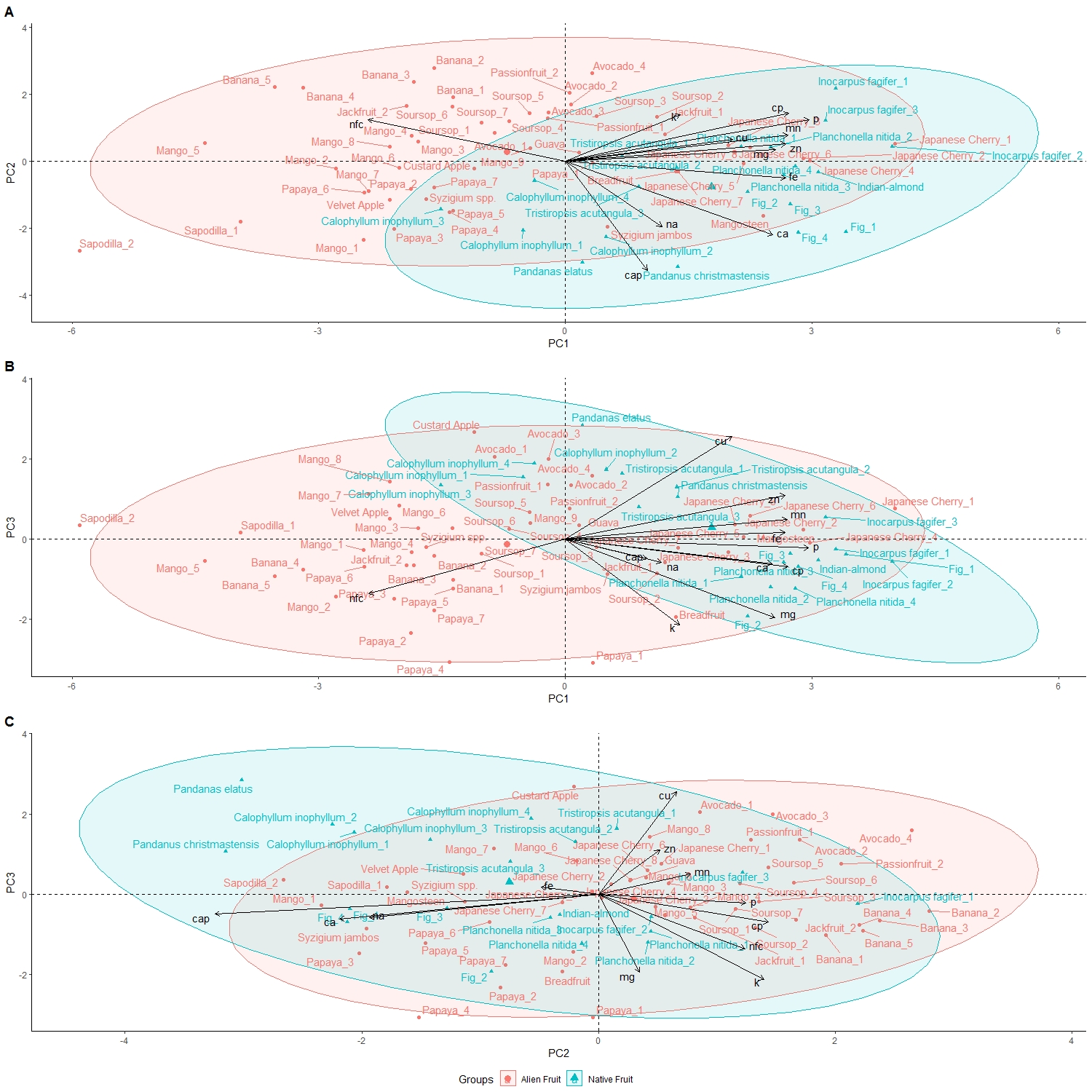

Supplement: S2 Fig — Results of the PCA for A) principle component (PC) 1 and PC 2, B) PC 1 and 3, and C) PC 2 and 3. (JPEG) [file pone.0250857.s002.jpeg]
